# Data on the calcium-induced mobility shift of myristoylated and non-myristoylated forms of neurocalcin delta

**DOI:** 10.1016/j.dib.2016.03.021

**Published:** 2016-03-11

**Authors:** Jeffrey Viviano, Anuradha Krishnan, Hao Wu, Venkat Venkataraman

**Affiliations:** a Graduate School of Biomedical Sciences, Rowan University, Stratford, NJ 08084, USA; b School of Osteopathic Medicine, Rowan University, Stratford, NJ 08084, USA

## Abstract

This data article presents the differences observed between the myristoylated and non-myristoylated forms of the neuronal calcium sensor protein, neurocalcin delta (NCALD). Analysis of the myristoylated and non-myristoylated versions of the protein by mass spectrometry provided difference in mass values consistent with addition of myristoyl group. In the presence of calcium, mobility retardation was observed upon electrophoresis of the protein in native gels. The retardation was dose-dependent and was exhibited by both the myristoylated and non-myristoylated forms of the protein.

## Specifications Table

**Table:** 

Subject area	*Biology*
More specific subject area	*Electrophoretic Techniques*
Type of data	*Table*, *graph*, *figure*
How data was acquired	*Mass spectroscopy*: Bruker Microflex LRF MALDI-TOF
*Electrophoresis*: *Bio-Rad miniPROTEAN*
Data format	*Analyzed*
Experimental factors	*For mass spectrometry*, *the samples were co-crystallized in 1:1 mixture of sinapinic acid and matrix solution containing acetonitrile*, *water and trifluoroacetic acid*.
*For electrophoresis*, *standard protocols were used.*
Experimental features	*Myristoylated and non-myristoylated forms of NCALD were analyzed*
Data source location	*Stratford*, *NJ 08012*, *USA*
Data accessibility	*Data is within this article*

**Value of the data**

• *The relevance of myristoylation to the biological functions of Neuronal Calcium Sensor* (*NCS*) *proteins remains to be elucidated.*
• *Data is presented on the myristoylated and non-myristoylated forms of NCALD assessed by calcium-induced mobility shift assay.*
• *The calcium-induced mobility shift assay may be useful in assessing the contribution of myristoylation and other post-translational modifications to response of all NCS proteins to calcium.*

## 1 Data

Bacterially expressed NCALD was purified in its myristoylated and non-myristoylated forms independently. Mass spectrometric analyses was carried out to determine the mass of each form and to determine if the myristoylated form also contained the non-myristoylated form. A table of the mass values of the two forms is provided, along with those documented in an earlier report by other investigators. Based on the analyses, there is little non-myristoylated NCALD in the myristoylated preparations. The two preparations were subjected to electrophoresis in native gels in the presence of incremental concentrations of calcium. A dose-dependent mobility retardation is observed with both forms. However, the myristoylated form exhibits a greater amplitude and increased sensitivity to change in calcium concentrations.

## 2 Experimental design, materials and methods

NCALD was expressed in *E. coli* ER2566 as described in [1]. Briefly, cells grown overnight were inoculated (1% inoculum) into fresh LB medium and grown to an optical density of 0.6 at 600 nm. IPTG (1 mM final concentration) was then added for induction. For myristoylation, cells with yeast *N*-Myristoyl Transferase were used and myristic acid was supplemented. Cells without the transferase were used to generate the non-myristoylated form; the supplementation was also skipped. Cells were collected 2.5 h after induction, sonicated and the protein was purified on phenyl sepharose columns as described previously [2], [3]. The purified protein was then washed with calcium-depleted Tris–Cl (20 mM; pH 7.5) to remove any residual calcium. Calcium removal was through the use of Chelex-100 resin (BioRad Laboratories, CA, USA) using standard procedures. For mass spectrometry, protein samples were co-crystallized with a 1:1 mixture of sinapinic acid and matrix solution (50% acetonitrile/0.05% trifluoroacetic acid in water). Mass spectrometric analyses were carried out in linear, negative modes on a Bruker LRF MALDI-TOF instrument. The mass values were in good agreement with those reported earlier for the respective forms [4] (see Table 1). There was no peak corresponding to the non-myristoylated form in the myristoylated preparation.

**Fig. 1:**
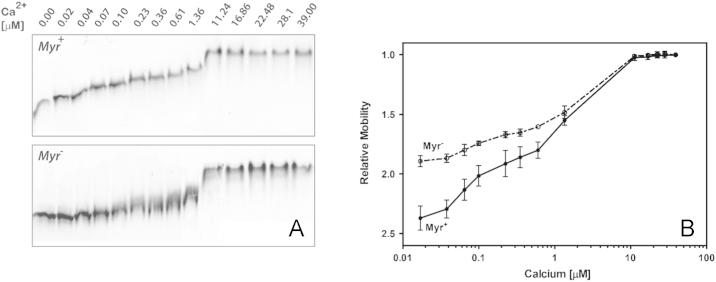
Effect of Myristoylation on Calcium-induced Mobility Shift on NCALD.

Mobility Retardation of NCALD (and other NCS proteins) in native gels has been documented. The retardation was directly dependent on the concentration of calcium. In order to determine if myristoylation of NCALD was essential for the calcium-dependent mobility shift, analyses were carried out with the non-myristoylated (Myr^−^) or myristoylated (Myr^+^) NCALD. Protein was incubated in the presence of indicated concentration of calcium using calibration buffers and electrophoresed in native gels as described [1]. A representative image of the gel is presented in Panel A (Fig. 1). Relative mobility values were determined from at least three experiments and plotted as a function of calcium concentration (Fig. 1; Panel B). To facilitate direct comparison, the data for the myristoylated NCALD has been reproduced from [1].

## Figures and Tables

**Table 1:** Mass Spectrometric Analyses of NCALD.

**Description**	**Myr**^**−**^**Neurocalcin**	**Myr**^**+**^**Neurocalcin**
Molar mass (g mol^−1^±SD)	22,107.1±0.8	22,325.8±1.4
(MALDI-MS)
Previously reported molar mass (g mol^−1^±SD)	22,110±2	22,325±2
(ESI-MS)
