# Supplementary material for: Data on the calcium-induced mobility shift of myristoylated and non-myristoylated forms of neurocalcin delta
Source: Data Brief. 2016 Mar 11;7:630–3. doi: 10.1016/j.dib.2016.03.021 (PMC4802544; doi:10.1016/j.dib.2016.03.021)
Supplement: Supplementary file 1 — Supplementary material [file mmc1.doc]

Conflict of Interest: None
